# Effect of Cassava Bioethanol By-Products and Crude Palm Oil Feeding on Fatty Acid Composition of Beef Meat and Fat in Crossbred Thai Indigenous Heifers

**DOI:** 10.3390/ani14233478

**Published:** 2024-12-02

**Authors:** Chirasak Phoemchalard, Ronnachai Prommachat, Tanom Tathong, Suthipong Uriyapongson

**Affiliations:** 1 Department of Agriculture, Mahidol University, Amnatcharoen Campus, Amnatcharoen 37000, Thailand; chirasak.pho@mahidol.edu; 2 Excellence Center on Agriculture and Food for Health, Mahidol University, Amnatcharoen Campus, Amnatcharoen 37000, Thailand; 3 Department of Animal Science, Faculty of Agriculture and Natural Resources, Rajamangala University of Technology Tawan-ok, Chonburi 20110, Thailand; ronnachai_pr@rmutto.ac.th; 4 Department of Food Technology, Faculty of Agriculture and Technology, Nakhon Phanom University, Nakhon Phanom 48000, Thailand; tanomi@ms.npu.ac.th; 5 Department of Animal Science, Faculty of Agriculture, Khon Kaen University, Khon Kaen 40002, Thailand

**Keywords:** fatty acid profiles, chemometrics, crude palm oil, by-products, beef, sustainability

## Abstract

The quality and nutritional value of meat are significantly attributed to the composition of fatty acids (FAs). This investigation used gas chromatography to assess FAs in *longissimus et lumborum* (LL), *semimembranosus* (SM), and subcutaneous fat (SC) tissues of 18 heifers feeding low (15%, LCEP) or high (30%, HCEP) cassava bioethanol by-products (CEP) and 0 (CPO-0), 2 (CPO-2), or 4% (CPO-4) crude palm oil (CPO). The experimental diet was provided at 1.75% of body weight, along with free access to rice straw and water for 150 days. The results showed that the highest content of saturated (SFAs, 50.14, 42.76, and 68.76%, mainly C16:0), monounsaturated (MUFAs, 44.89, 49.14, and 30.41%, mainly C18:1n9c), and polyunsaturated fatty acids (PUFAs, 4.96, 8.10, and 0.84%, mainly C18:2n6c and C18:2n6t) were observed in LL, SM, and fat tissues. CPO supplementation significantly affected the FAs in LL and SM meat, with CPO-2 and CPO-4 diets leading to decreased SFAs and increased MUFAs and PUFAs compared to the CPO-0 diet. Multivariate analysis showed the most important FAs that highlight discrimination between different oil supplementation levels (CPO-0 vs. CPO-2, CPO-0 vs. CPO-4, CPO-2 vs. CPO-4) in LL (C18:2n6c, C20:3n3, C13:0), SM (C13:0, C18:0, C13:0), and SC fat (C18:2n6t, none, none) tissues. This data generates key insights into FA profiles resulting from different levels of oil supplements in cattle diets, which could influence future research on precision nutrition in beef production.

## Simple Summary

This study determined whether cassava bioethanol by-products (CEP) and crude palm oil (CPO) alter fatty acid profiles and lipid quality indices of beef from Thai indigenous crossbred heifers. Supplementation with CPO resulted in fatty acid profiles that differed significantly in loin and round meat compared to diets without CPO; it decreased saturated fatty acids and increased unsaturated fatty acids. The interaction of CEP and CPO has been found to modulate levels of certain fatty acids in subcutaneous fat. CPO-fed beef also improved some lipid quality indices. Successful chemometric analysis discriminated between CPO levels using fatty acid profiles. Based on these findings, dietary manipulation by CEP and CPO can be used to affect the fatty acid composition and nutritional quality of beef for the benefit of consumers.

## 1. Introduction

The composition of fatty acids in beef meat greatly influences its nutritional quality, organoleptic properties, and consumer acceptance [1,2,3,4]. With the demand for better beef products increasing in the international markets, there is more incentive to develop better feeding regimes that improve the fatty acid composition of beef meat [5,6,7]. Strategic diet manipulation is crucial for optimizing meat’s fatty acid composition, enhancing health benefits for consumers [7,8]. The type of feed—whether it be forage-based, grain-based, or oil-based—directly alters the fatty acid profile in livestock [9,10,11,12].

Crude palm oil (CPO) serves as a vital component in animal nutrition, providing a high energy content, essential fatty acids, and vitamins that enhance livestock health and productivity [13,14]. Cassava, a starch-rich crop widely used in bioethanol production [15,16], yields cassava bioethanol by-products (CEP) with potential as animal feed [17,18]. Utilizing the bioethanol industry by-products can improve the environmental sustainability and cost-effectiveness of beef production systems. Previous research has examined the effects of CEP and CPO on nutrient digestibility, growth performance [17], carcass characteristics, and meat quality [18] in heifers. However, their impact on fatty acid profiles across different tissues remains unexplored.

This research, therefore, investigates the fatty acid composition of *longissimus et lumborum* (LL), *semimembranosus* (SM), and subcutaneous fat (SC) in crossbred beef heifers fed cassava bioethanol by-products and crude palm oil. Additionally, chemometric analysis was used to identify fatty acid biomarkers in each tissue between feeding levels. In this study, we will elucidate how these dietary interventions influence fatty acid profiles in beef meat, which will be useful to the beef industry and researchers looking to optimize beef production systems for quality and sustainability. The study hypothesizes that the fatty acid composition of beef meat and subcutaneous fat tissue from heifers differs from that of beef, affecting their nutritional profiles. It also seeks to identify potential fatty acid biomarkers in response to different levels of dietary oil supplementation.

## 2. Materials and Methods

All animal procedures followed the National Research Council of Thailand’s Ethical Principles and Guidelines for the Use of Animals and received approval from Khon Kaen University’s Animal Care and Use Committee (approval number: AEKKU 77/2556).

### 2.1. Animal and Diet Background and Meat Samples

Eighteen yearling Brahman–Thai native crossbred heifers (130 ± 14 kg BW, 1-year-old) were used. They received a concentrate feed at 1.75% BW, with free access to water and rice straw for 5 months. The treatments consisted of two levels of cassava bioethanol by-product (15 or 30%; LCEP or HCEP) each supplemented with three levels of crude palm oil (0, 2, and 4%; CPO-0, CPO-2, and CPO-4), designated as (1) LCEP + CPO-0, (2) LCEP + CPO-2, (3) LCEP + CPO-4, (4) HCEP + CPO-0, (5) HCEP + CPO-2, and (6) HCEP + CPO-4, respectively. Growth performance, nutrient digestibility, carcass traits, and meat quality results have been reported in detail elsewhere [17,18]. In brief, the results demonstrated that feed intake was exclusively influenced by the concentration of CPO, with elevated CPO levels enhancing fat digestibility. Nutrient intake and growth performance were similar. The DM, CP, and EE digestibility of HCEP was lower than LCEP [17]. The lean meat percentage and meat/bone ratio were less than those of LCEP-fed cattle. Carcass fat, fat content, and meat redness were significantly increased with diets with 4% dietary CPO [18]. After measurement of carcass characteristics, around 100 g of each *longissimus et lumborum* (LL) and *semimembranosus* (SM) from the right side of the carcass, and 25 g of subcutaneous fat (SC) at the 12th and 13th rib were then collected (within 1 h p.m.), packed in an LDPE vacuum bag, and frozen in the freezer at −25 °C for six months before examining fatty acid profiles.

### 2.2. Analysis of Fatty Acid Profiles

Total lipids are extracted using chloroform–methanol in feed, LL, SM, and SC fat samples [19,20]. The 15 g of ground samples were extracted with 90 mL of chloroform-methanol (2:1 *v*/*v*) and blended for 120 s, then 30 mL of chloroform was added and blended again for 120 s using Nissei AM-8 Homogenizer (Nihon Seiki Kaisha, Ltd., Tokyo, Japan). The homogenates were isolated in the separating funnel, followed by an additional 30 mL of dH_2_O and 5 mL of 0.58% NaCl, then shaken and set aside before clear separation of the solution. The lower solution was left to the known weight of the evaporate flask. The solvent is extracted from fat by BUCHI Rotavapor R-200 Rotary Evaporator (BUCHI Labortecnnik AG, Flawil, Switzerland), evaporating at 40 °C and then deposited at −20 °C under N_2_ gas. The extracted lipids were methylated to yield fatty acid methyl esters (FAMEs) [21]. At first, 30 mg of the extracted lipids were transferred to a 15 mL test tube with a screw cap. Next, 1.5 mL of 0.5 N NaOH/MeOH was applied, flushed by N_2_, covered, heated for 5 min at 100 °C through intermittent shaking, and then kept cool at room temperature. Then, 1 mL of heptadecanoic acid (C17:0), as an internal standard, was added, and 2 mL of 14% BF_3_/MeOH, heated for 5 min at 100 °C by infrequent trembling, and 10 mL of dH_2_O then added. Each methylated solution was placed in a centrifuged tube, and the mixture with hexane (5 mL) was then centrifuged at a speed of 5000× *g* for 15 min. The hexane layer was completely dehydrated by sodium sulfate (Na_2_SO_4_), transferred to amber vial, flushed by N Rapid Preparation of Fatty Acid Esters from Lipids for Gas Chromatographic Analysis, and FAMEs was analyzed by gas chromatography (GC) (HP 6890 GC Systems, Agilent Technologies, Inc., Wilmington, DE, USA) fitted with a capillary column (SP-2560, L × I.D. 100 m × 0.25 mm, 0.20 μm film thickness). The settings of the GC conditions were as follows: carrier gas, He, injector temperature, 250 °C, flame ionization detector (FID), 260 °C, split ratio, 100:1, oven temperature, for 5 min at 140 °C, and the temperature was then raised to 240 °C at a rate of 4 °C/min. Each 1 μL of the sample was automatically injected, the fatty acid peaks were defined and compared to established reference methyl esters. All fatty acid values were calculated as a percentage of total fatty acids by weight. Total saturated fatty acids (SFA), monounsaturated fatty acids (MUFA), polyunsaturated fatty acids (PUFA), PUFA/SFA ratio (P/S), n-3 and n-6 fatty acids, and n-6/n-3 ratio were calculated. Other lipid quality indices were also computed.

### 2.3. Analysis of Healthy Indices of Lipids

The various quality indices of lipids were calculated to determine their atherogenicity index (AI), thrombogenicity index (TI), hypocholesterolemia/hypercholesterolemic ratio (h/H), nutritive value index (NVI), health-promoting index (HPI), Peroxidizability index (PI), and the enzyme activity index were also computed.

#### 2.3.1. Atherogenicity Index (AI)

The AI highlights the balance between pro-atherogenic saturated fatty acids and anti-atherogenic unsaturated fatty acids. These fatty acids contribute to the development of immune cells and the circulatory system. AI was calculated using the following formula [22]:

$$\mathrm{AI}=\frac{\left[C12:0+(4\times C14:0)+C16:0\right]}{\left(n-3PUFA+n-6PUFA+MUFA\right)}$$

#### 2.3.2. Thrombogenicity Index (TI)

The TI indicates the tendency of the blood vessel to form clots. It is linked between prothrombogenetic (saturated) and anti-thrombogenetic (unsaturated) fatty acids as per the following formula [23].

$$\mathrm{TI}=\frac{\left[C14:0+C16:0+C18:0\right]}{\left[(0.5\times \Sigma MUFA)+(0.5\times \Sigma PUFA)+(0.5\times n-6PUFA)+(3\times n-3PUFA)+(n-3PUFA/n-6PUFA)\right]}$$

#### 2.3.3. Hypocholesterolemic/Hypercholesterolemic Ratio (h/H)

The h/H ratio is a commonly used index for the fatty acid profile of meat and blood plasma. It shows the relationship between the observed hypocholesterolemic (unsaturated) fatty acid and the hypercholesterolemic (saturated) fatty acids as per the following formula [24].

$$h/H=\frac{\left(C18:1+C18:2+C18:3+CLA+C20:2+C20:3+C22:6\right)}{\left(C14:0+C16:0\right)}$$

#### 2.3.4. Nutritive Value Index (NVI)

The NVI values were calculated as per the following equation [25]:

$$\mathrm{NVI}=\frac{\left(C18:0+C18:1\right)}{\left(C16:0\right)}$$

#### 2.3.5. Health-Promoting Index (HPI)

The HPI evaluates the nutritional quality of various dietary fats by examining how their composition impacts cardiovascular disease (CVD) [26].

$$\mathrm{HPI}=\frac{\Sigma UFA}{\left[C12:0+(4\times C14:0)+C16:0\right]}$$

#### 2.3.6. Peroxidizability Index (PI)

The PI values was calculated as following equation [27]:

$$\begin{array}{c} PI=(monoenoic\;acid\times 0.025)+(dienoic\;acid\times 1)+(trienoic\;acid\times 2)\;+\\ (tetraenoic\;acid\times 4)+(pentaenoic\;acid\times 6)+(hexaenoic\;acid\times 8). \end{array}$$

#### 2.3.7. Enzyme Activity Index

The delta-9 fatty acid desaturation index was used as an indicator of metabolic disease [28].

$$\begin{array}{c} \text{-}\Delta 9\text{-}desaturase\;16\;index\;\left(SCD\text{-}16\right)\\ SCD\text{-}16=\frac{\left[C16:1\right]}{\left(C16:0+C16:1\right)}\times 100\\ \text{-}\Delta 9\text{-}desaturase\;18\;index\;(SCD\text{-}18)\\ SCD\text{-}18=\frac{\left(C18:1\right)}{\left(C18:0+C18:1\right)}\times 100\\ \text{-}Elongase\;index\;(EI)\\ EI=\frac{\left(C18:0+C18:1\right)}{\left(C16:0+C16:1+C18:0+C18:1\right)}\times 100\\ \text{-}Thioesterase\;(TE)\\ TE=\frac{\left(C16:0\right)}{\left(C14:0\right)} \end{array}$$

### 2.4. Statistical Analysis

Data is presented as least square means ± SD. Fatty acid content, lipid indices, and enzyme indexes were analyzed for analysis of variance (ANOVA) using 2 × 3 factorial arrangements in a completely randomized design (CRD) with a GLM procedure of SAS [29]. The analysis included two levels of CEP and three levels of CPO as main factors, along with their interaction (CPO × CEP). The differences among means were compared using Tukey–Kramer post hoc test (*p* < 0.05). A Venn diagram was created with a free online tool (https://bioinformatics.psb.ugent.be/webtools/Venn/, accessed on 23 September 2024). Multivariate analysis was performed using the free online tool, MetaboAnalyst 6.0 [30]. Principal components analysis (PCA) was initially used for visualization. Orthogonal partial least squares discriminant analysis (OPLS-DA) was then conducted to identify candidate biomarkers based on variable importance in projection (VIP > 1) values, *p*-Values (*p* < 0.05), and false discovery rate (FDR) (*p* < 0.05) [31,32].

## 3. Results

### 3.1. Fatty Acid Profiles

Fatty acid profiles (% of total FA) of experimental diets are shown in Table 1. The percentages of fatty acid profiles in meat and fat tissues obtained from beef heifers fed diets with different levels of CEP and CPO are displayed as least square means and standard deviations in Table 2, Table 3 and Table 4. In both LL and SM meat, the interaction effect between CEP and CPO and the main effect of CEP was not significant (*p* > 0.05), indicating there were no significant differences for any fatty acid profiles for each factor level combination of CEP and CPO or CEP alone.

For LL meat, the main effect of CPO was significant (*p* < 0.05), affecting the levels of several fatty acids, including C12:0, C13:0, C14:0, C14:1, C16:0, C16:1, C18:0, C18:1n9t, C18:1n9c, C18:2n6c, C20:3n6, C20:3n3, C22:6n3, as well as SFAs, MUFAs, PUFAs, omega-3, omega-6, omega-6:3, PUFA:SFA, MUFA:SFA, UFA:SFA, and HFA:SFA. The concentrations of C12:0, C14:0, C14:1, C16:0, C18:0, C18:1n9t, SFAs, omega-6:3, and HFA:SFA were significantly higher in the CPO-0 group compared to the CPO-2 and CPO-4 groups. The levels of C14:1, C18:1n9ct, C18:1n9c, and HFA:SFA were significantly higher in the CPO-2 group than in the CPO-4 group. While the values of C13:0, C16:1, C18:2n6c, C20:3n6, C20:3n3, C22:6n3, MUFAs, PUFAs, omega-3, omega-6, PUFA:SFA, MUFA:SFA, and UFA:SFA for CPO-0 were significantly smaller than those of CPO-2 and CPO-4. There was no significant difference in C18:3n3 across CPO levels.

Including CPO in the heifer diet, notably impacts the fatty acid profiles of SM meat (*p* < 0.05, Table 3). The CPO-2 diet resulted in lower levels of saturated (C12:0, 14:0, 16:0, 18:0) and some unsaturated (14:1, 18:1n9t, 18:2n6c, 18:3n3) fatty acids, together with total SFAs, PUFAs, omega-6, omega-6:3, and PUFA:SFA than heifers consuming CPO-0 and CPO-4 diets. In opposition, it increased C13:0, C16:1, C18:1n9c, C20:3n3, C22:6n3, total MUFAs, omega-3, MUFA:SFA, UFA:SFA, and HFA:SFA. C20:3n6 remained unaffected by CPO levels.

The levels of C16:1, C18:2n6c, C20:3n6, and omega-6:3 in SC fat (Table 4) displayed a statistically significant interaction between CEP and CPO (*p* < 0.05). The mix of low CEP (LCEP) and CPO-0 led to lower C16:1, C18:2n6c, and omega-6:3 levels than those of other groups. The LCEP and CPO-4 combination had the lowest C20:3n6. Furthermore, both LCEP and HCEP affected fatty acids C10:0, C9T11, C18:3n3, C9C11, C21:0, C22:6n3, PUFAs, omega-3, omega-6, and PUFA:SFA (*p* < 0.05), with the LCEP group presenting higher amounts of C10:0 and C18:3n3, and lower values of C9T11, C9C11, C21:0, C22:6n3, PUFAs, omega-3, omega-6, and PUFA:SFA compared to the HCEP group. The SC fat from heifers whose diet included CPO revealed effect on C10:0, C13:0, C16:0, C18:2n6t, C20:3n3, and C22:6n3 (*p* < 0.05), observing that the CPO-0 group had higher C10:0, C13:0, C18:2n6t, and C20:3n3 levels than those of CPO-2 and CPO-4 groups. In contrast, C16:0 and C22:6n3 in the CPO-4 group were greater than those in the CPO-2 group. No discernible distinctions were found for C12:0, C14:0, C18:0, C18:1n9c, T10C12, C20:2, SFAs, or MUFAs among various CEP or CPO levels.

### 3.2. Healthy Indices of Lipids

Quality indices of lipids in LL, SM meat, and SC fat under different levels of CEP and CPO are shown in Table 2 and Table 3. For lipid quality indices, there was no significant interaction (*p* < 0.05) between CEP and CPO in both meats, and the main effect of CEP was not observed. Nevertheless, CPO pronounced impacted various indices in LL meat (*p* < 0.05) such as AI, TI, h:H, NVI, HPI, PI, SCD-16, SCD-18, EI, and TE. Supplementation with a higher CPO level in the diet resulted in reduced indices of AI, TI, and EI, but increased h:H, NVI, HPI, PI, SCD-16, SCD-18, and TE. Similarly, CPO-2 improved h:H, NVI, HPI, SCD-16, SCD-18, and TE, but had lowered AI, TI, and EI compared to the other dietary treatments in SM meat (Table 2 and Table 3). A significant CEP x CPO interaction was seen for SCD-16 in SC fat (*p* < 0.05). The HCEP combined with the CPO-4 group containing the highest SCD-16 value. Nonetheless, it was similar to other treatments with the exception of LCEP and CPO-0. There were no significant differences between the LCEP and HCEP groups for any CPO level in the AI parameter. Similarly, the TI, h:H, NVI, HPI, and EI were not significantly different among treatments. The PI and TE index were different between the LCEP and HCEP groups *(p* < 0.05).

### 3.3. Multivariate Analysis Using Chemometric Approach

The PCA and OPLS-DA (Figures S1–S3) score plots were used to analyze the percentages of FAs. The principal components of PC1 and PC2 explained 88.7% and 9.9% of the variation in LL, 68.5% and 24.2% in SM, and 74.0% and 11.9% in SC fat, respectively. However, the CPO groups were not entirely separated in the unsupervised PCA of SC fat tissues. Based on OPLS-DA models (Table S1 and Figure S1), LL with CPO-0 versus CPO-2 were discriminated with a model with a R^2^X of 0.82, R^2^Y of 1.00, a Q^2^ of 0.98, and a VIP threshold of 1.0 with *p*-Value and FDR less than 0.05 (Table S1). In this model, 7 FAs including C18:2n6c, C13:0, C14:0, C20:3n3, C12:0, C22:6n3, and C16:0 were found to carry the class separation. Clear separation of LL with CPO-0 from CPO-4 with a model including 12 FAs such as C20:3n3, C20:3n6, C14:0, C16:0, C12:0, C18:2n6c, C13:0, C22:6n3, C18:1n9t, C18:0, C16:1, and C14:1 (R^2^X of 0.97, R^2^Y of 1.00, Q^2^ of 1.00) were observed. OPLS-DA model also showed a clear separation of CPO-2 vs. CPO-4 with a model including 8 FAs including C13:0, C14:1, C20:3n6, C18:3n3, C16:1, C14:0, C12:0, and C16:0 with a R^2^X of 0.79, R^2^Y of 1.00, a Q^2^ of 0.98.

In SM meat (Table S1 and Figure S2), 8 FAs including C13:0, C14:1, C20:3n6, C18:3n3, C16:1, C14:0, C12:0, and C16:0 were the potential FAs that could distinguish between CPO-0 versus CPO-2 groups. The key parameters of this model, R^2^X = 0.79, R^2^Y = 1.00, and Q^2^ = 0.98, were good predictors of model fit. The OPLS-DA model showed good performance in discriminating between CPO-0 and CPO-4, with 8 FAs including C18:0, C22:6n3, C13:0, C16:1, C18:1n9t, C18:1n9c, C18:3n3, and C20:3n3 were the key discrimination (R^2^X = 1.00, R^2^Y = 1.00, and Q^2^ = 1.00). Lastly, good discrimination was shown between CPO-2 and CPO-4 with C13:0, C18:1n9c, C18:1n9t, C18:3n3, C18:2n6c, C12:0, C14:1, with a R^2^X = 0.89, R^2^Y of 1.00, a Q^2^ of 0.99. In fat tissues (Table S1 and Figure S3), only the single most identifying variable (C18:2n6t) was prioritized for comparing CPO-0 with CPO-2 (R^2^X = 0.63, R^2^Y of 1.00, a Q^2^ of 0.94). While there were no significant findings to identify the key FAs for distinguishing between CPO-0 vs. CPO-4 and CPO-2 vs. CPO-4.

**Figure 1:**
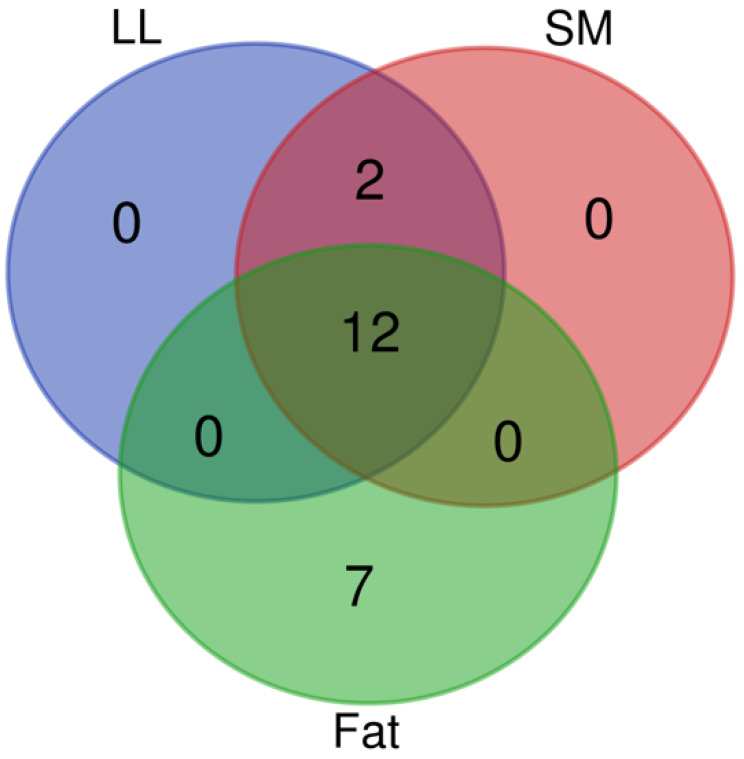
Venn diagram of fatty acid profiles. The Venn diagram depicts the fatty acids shared by LL meat, SM meat, and SC fat of Brahman crossbred heifers fed varied diets. The overlapping sections denote the number of fatty acids shared by the tissues shown by the circles, which each reflect the total number of fatty acids contained in their respective tissues.

A Venn diagram (Figure 1) is a graphic representation that makes use of concentric circles to highlight the connections between different things or limited sets of things. The characteristics shared by overlapping circles are not shared by nonoverlapping ones. A total of 21 components contributed to this effort, and LL, SM, or fat influenced the overflow at the same time. The 14 parameters listed below were measured at multiple locations: For LL and SM, C14:1 and C18:1n9t were measured on the plane, while the other 12 parameters (C12:0, C13:0, C14:0, C16:0, C16:1, C18:0, C18:1n9c, C18:2n6c, C18:3n3, C20:3n3, C20:3n6, and C22:6n3) were measured on each of the three planes. So, the 7 separate variables (C10:0, C9T11, C9C11, T10C12, C18:2n6t, C20:2, and C21:0) were measured based on fat alone.

## 4. Discussion

Concerning human nutrition, red meat is an important source of essential polyunsaturated fatty acids (PUFAs), protein, vitamins, and minerals [6,33]. Nonetheless, feeding regimens practiced in beef cattle production can modify the fatty acid composition of their meat and, thus, it could also influence the content of essential fatty acids [8,12]. For instance, linseed oil supplementation promotes n-3 PUFA and CLA deposition and improves the n-6/n-3 PUFA ratio [34]. The fatty acids found in the intramuscular fat (IMF) of beef typically contain around 45 to 48% saturated fats and between 35 and 45% monounsaturated fats, with a slight percentage of polyunsaturated fats (5%) [6]. Results from our investigation indicate that increasing CPO levels linearly reduced SFAs in LL (55.62, 52.18, and 42.61%). However, in SM, the lowest SFA content was observed in the CPO-2 group. Changes in SFAs are caused by biohydrogenation of dietary unsaturated fat by rumen bacteria [35,36]. With high-concentrate diets, biohydrogenation of PUFA in the rumen is reduced [37] and causes the unsaturated deposition. In LL, higher CPO levels linearly increased in both MUFAs (41.12, 43.60, and 49.95%) and PUFAs (3.25, 4.21, 7.42%). In SM, MUFAs content was higher in CPO-2 (51.66%) compared to CPO-4 (47.90%) and CPO-0 (47.85%). Additionally, SM PUFA content was lower in CPO-supplemented groups (7.93 and 7.86%) than in the CPO-0 (8.49%). Compared to other palm oil studies in ruminants, no changes in the total fatty acid composition were found when Friesian bulls were fed tallow or various hydrogenated palm oils at 5.5% [38] or Friesian steers fed different types of fats at 4% [39]. In SC fat, PUFA content was affected only by CEP level, with HCEP showing a higher value (0.95%) than LCEP (0.73%). The values are consistent with the grain-fed beef report in the US, where SFAs, MUFAs, and PUFAs averaged 43.4, 45.3, and 4.5%, respectively [40]. These fatty acid profiles in meat are important because they alter both its nutritional content and sensory properties, such as juiciness and taste [7].

Among dietary SFAs, myristic (C14:0) and palmitic (C16:0) acids are known to elevate plasma cholesterol, with myristic acid exerting a stronger effect. In contrast, stearic acid (C18:0) has minimal impact on cholesterol levels [41,42]. Our findings reveal a fatty acid profile of C16:0 > C18:0 > C14:0, in which the higher CPO increased C16:0 and C18:0 in SC fat, while the values in LL and SM were opposite. The greater proportion of C16:0 than C18:0 and C14:0 are consistent with previous reports on African beef [11], US beef [40], young bull [43], Brahman steers [44], and Korean native cattle [45]. Regarding MUFAs, our analysis revealed that oleic acid (C18:1n9c) is the predominant fatty acid, constituting 28.5% of total fatty acids, followed by palmitoleic acid (C16:1). Oleic acid proportions differed significantly amongst samples at different CPO levels, where the values in LL and SM reduced with higher dietary fat levels. This agrees with previous studies on Nellore steers, where oleic acid concentrations were lowest in steers fed diets with supplemental lipid sources [12]. This MUFA profile aligns with previous findings in young beef cattle fed with grass or grain diets [11], conventional beef [46], and fat-fed beef [39,44], where oleic acid is consistently the most abundant MUFA. The oleic acid content in food not only contributes to its palatability but also offers potential health benefits [47,48], particularly lowering LDL cholesterol.

PUFAs are recognized in beef for their cholesterol-lowering properties and various health benefits [47]. Our findings indicated that PUFA levels varied significantly in beef samples, spanning from 0.84% in fat to 4.96% in LL and 8.10% in SM meat. The PUFA content in LL increased proportionally with CPO supplementation levels. However, in SM, the content was lower in treatments with higher CPO levels compared to CPO-0. Among PUFAs, the main n-6 fatty acid in all samples was linoleic acid (C18:2n-6c), followed by C20:3n3 (ETE) and C22:6n3 (DHA). Similar PUFAs in loin and SC fat were observed in Nellore steers fed varied lipid sources [12]. However, palm kernel oil supplementation did not affect PUFAs in bulls [43]. These results correlate with investigations from Africa, Argentina, and the UK that identified greater levels of n-6 fatty acids in beef produced by grain-fed cattle than from grass-fed cattle [11,49,50].

The proportion of conjugated linoleic acid (CLA) in beef affects its nutritional quality and health benefits [7]. Dietary supplementation with CLA reduces body fat, increases lean muscle mass, decreases atherosclerosis severity, and inhibits carcinogenesis [51,52,53]. We found that only CLA isomers (C9T11, C9C11, T10C12) existed in SC fat but not in LL and SM meat, and the C9T11 and C9C11 levels increased with HCEP diets. Several studies have shown that cattle fed on a forage-rich diet, such as grass, can increase CLA levels in meat [10,50,54].

Thrombotic and atherogenic indices are managed by omega 3 and omega 6 fatty acids, and their balance affects the hypocholesterolemic index [55]. Decreases in the omega 6 ratio typically decrease inflammation and elevate long-term illness risks [56]. A reduced omega 6 to omega 3 ratio is better for lowering the chances of numerous chronic illnesses prevalent in several nations [57]. In Western diets, the ratio normally stands at around 16:1; however, a 1:1 ratio is viewed as the best option [58,59,60]. In the current study, dietary CPO linearly increased omega 3 (1.47, 2.00, 3.52) and omega 6 (1.79, 2.21, 3.90) but decreased omega 6:3 ratio (1.23, 1.10, 1.11) in LL. However, in SM, the lowest omega 3 was found in CPO-4, while the lowest omega 6 and omega 6:3 ratio was found in CPO-2. In SC fat omega 3 and 6 of HCEP were higher than LCEP. Additionally, heifers fed HCEP and CPO-4 had the lowest omega 6:3 ratio (3.90) compared to other treatments. Previous works have shown that different cattle breeds and dietary fat supplementations have yielded different omega 6:3 ratio. According to Fiorentini et al. [12], Nellore steers fed various lipid sources had ratios varying from 4.50 to 12.20. Supplementation of 5.5% of different fat types to Friesian steers resulted in higher ratios of 16.73–20.57 [39]. Bulls fed palm kernel oil from 0.0–34.6 g/kg diet had lower values of 2.54–2.22 [43]. The ratio was approximately 10.53 for Brahman steers supplemented with 200 g/day palm oil. In comparison, grass-fed beef generally has a more favorable fatty acid profile than grain-fed beef, with higher omega 3 PUFA content and lower omega 6:3 ratios [54]. Maximizing meat quality is challenging because very high PUFA levels may alter the content of these profiles [6]. Some enhancements in the fatty acid profile of beef can be achieved while still maintaining desired meat quality [8,61].

The recommended PUFA to SFA (P:S) ratios in foods is greater than or equal to 0.40 [62]. Levels lower than this threshold are considered undesirable for the human diet [63] because of their potential to increase blood cholesterol. In this study, P:S ratios of LL ranged from 0.08 in CPO-0 to 0.15 in CPO-4, SM ranged from 0.15 in CPO-0 to 0.18 in CPO-4, and SC ranged from 0.01 to 0.02 when comparing CPO-0 to CPO-4. Similar results are also reported by [3], where the P:S ratios in the lean beef from feeding a ruminally-protected lipid was 0.11, indicating a relatively high in SFA content. Also, this proportion in beef is generally minimal, approximating 0.1 [6].

Dietary CPO linearly increased the unsaturated to saturated fatty acid ratio (UFA:SFA) in LL meat (0.80, 0.92, and 1.35). Furthermore, our findings indicate that the CPO-2 diet significantly elevated the UFA:SFA ratio in both the SM meat and SC fat. The present results concord with another study that investigated the ratio of UFA:SFA in porcine meat generation and stated that the UFA:SFA ratio was 1.32 [64]. The primary determinant of meat flavor profiles is the UFA:SFA ratio, in which UFA has important roles in sensory taste enhancement as well as the removal of deleterious free radicals [65].

Potential cardiovascular health impacts are expressed as the ratio of hypercholesterolemic (HFA) to saturated fatty acids (HFA:SFA) in meat. The higher presence of HFA:SFA ratio indicates a greater risk of cardiovascular diseases [8,66]. In this study, beef from CPO-2 and CPO-0 treatment had higher HFA:SFA ratios when compared to CPO-4. The LL, SM, and fat from CPO-fed beef had the mean HFA:SFA ratios of 0.67, 0.67, and 0.69, respectively. The results reported in this work are similar to those in broiler meat (0.62) [67] and organic cow milk (0.74) [68].

Healthy indices of lipids are important tools for evaluating the nutritional quality and potential health impacts of fatty acids in meat. The assessment of lipid profiles in relation to cardiovascular health and overall nutritional quality involves several key indices. The atherogenicity (AI) and thrombogenicity (TI) indices evaluate the potential for atherosclerosis and thrombosis development, respectively [22]. Lower AI and TI indicate a decreased risk of developing cardiovascular diseases. This study reveals that AI (0.85), and TI (1.35) are approximately 3 times less in meat (LL and SM) than in SC fat (2.54 and 4.31). In LL meat, AI and TI were decreased by increasing the CPO levels, but in SM, the lowest values were presented in heifers fed CPO-2 and these values were similar in SC fat. However, the results obtained are greater than those for beef [69].

For the fatty acids in the diet, the hypocholesterolemic to hypercholesterolemic ratio (h:H) provides insight into cholesterol-lowering capacity, with a higher h:H being preferred in human health [27]. The h:H index obtained in the present study ranged from 0.91 to 1.38, which was lower than goose meat (2.7) [27], lamb (1.92) [70], and beef (1.8) [71]. The nutritive value index (NVI) is a ratio of the nutritional value of the meat, with a higher value indicating high nutritional value [25]. The highest value of NVI was noted for the SM meat (1.71) due to the highest proportion of C18:0, C 18:1, and the lowest C16:0 content. Similar results were found in hair and rabbit meat [72].

Regarding the main effect of CPO, HPI, and PI in LL linearly increased with increasing CPO levels. A higher value in the health-promoting index (HPI) indicates an association with a higher health benefit potential for human health [66]. Our study found HPI values of 1.26 for meat and 0.4 for fat. In comparison, other studies reported HPI ranges of 0.16 to 0.68 for milk [66] and 1.59 to 1.81 for poultry [73]. The peroxidizabilityi index (PI) measures the susceptibility of fatty acids to oxidative damage and its role in determining lipid stability as well as potential health impact, but as PI increases, the greater the protective potential against coronary artery disease [27]. The lower PI [11] of this present beef, compared to poultry meat [27], indicates fewer fatty acids autooxidation and longer shelf life.

The Δ9-desaturase enzymes, particularly SCD-16 and SCD-18, are crucial for con-verting SFAs into MUFAs. Higher activity of these enzymes is generally beneficial because it leads to increased production of palmitoleic acid (C16:1) and oleic acid (18:1n-9), which is known to be associated with improved lipid profiles and less inflammation [65,74]. In contrast, some research indicates that food with lower amounts of SCD-16 and SCD-18 tends to improve health outcomes because it decreases risks linked to metabolic syndrome and obesity [75,76,77,78]. In this experiment, the SCD-16 (25.42, 28.61, and 46.00) and SCD-18 (61.08, 64.04, and 66.74) values of LL meat rose as the CPO levels increased. But in SM, SCD-16 (41.95, 48.80, and 44.52) and SCD-18 (67.92, 68.91, and 65.01) showed the highest values for cattle-fed CPO-2 compared to others. The SCD-16 value was the lowest for SC from the LCEP and CPO-0 treatment compared to other groups. Among tissues, these enzymes were most abundant in SM > LL > fat. Compared to Nellore bulls fed 0–30% crude glycerin [79], where SCD-16 ranged from 8.5–13.6 and SCD-18 from 63.6–74.4 in loin, and SCD-16 from 9.4–14.1 and SCD-18 from 70.0–74.9 in fat, our results exhibit that CPO-fed cattle have nearly equal SCD-18 content in meat and fat, but higher SCD-16 in meat and lower SCD-16 in fat.

The elongase index (EI) and thioesterase index (TE) are also central to fatty acid metabolism. A higher EI may reflect an increased ability to desaturate fatty acids elongated via the elongase pathway [73], which is advantageous in generating long-chain FAs required for multiple physiological processes [28,76]. On the other hand, an excess of this elongation can be associated with the rise in SFAs and some negative effects on human health [80], so a moderate EI is preferable. The TE, depending on the activity of enzymes related to fatty acid synthesis, should also be optimal; low activity may be beneficial in protecting from excessive fat deposition, while high activity can lead to metabolic imbalances and health issues [81,82]. The reduced EI and TE indices of meat observed in this study may be explained by the increased conversion of palmitic and stearic acids to their respective unsaturated substances, possibly as a result of increased SCD-1 expression [67].

The OPLS-DA models constructed indicate an alteration in the fatty acid content of LL, SM, and SC fat because of oil supplementation. The clear classification between groups with different CPO content (0%, 2%, and 4%) demonstrates the effect of dietary fats on meat fatty acids with likely effects on its quality and nutrition. However, in terms of fat samples, evident separation was only achieved with 0 and 2% of CPO. The analysis highlighted some key FAs as important in the discrimination of treatment groups. Four FAs in LL (C12:0, C13:0, C14:0, and C16:0) and two FAs in SM (C13:0 and C18:3n3) were identified as key differentiators between 0, 2, and 4% of CPO. These key FAs can be useful in revealing the reasons behind the meat and health quality of animals with different diets. An OPLS-DA model with 7, 12, and 8 FAs, and 8, 8, and 7 FAs succeeded in separating LL and SM meat with 0 vs. 2, 0 vs. 4, and 0 vs. 4 CPO. Of these, Q^2^ values around 1 (higher than 0.9) indicate that the model was excellent [32,83]. Interestingly, while several key FAs distinguished CPO-0 and CPO-2 groups in meat, only one FA (C18:2n6t) was significant in fat tissues. The proposed 0 vs. 4 and 2 vs. 4 observed the same low oil restriction, indicating that more oil levels may not distinguish fatty acid compositions on top of what has already been achieved with lower levels.

## 5. Conclusions

It could be concluded that crude palm oil (CPO) supplementation in heifer diets greatly improves beef fatty acid profiles and nutritional quality. Both *longissimus lumborum* (CPO-4) and *semimembranosus* (CPO-2) muscles reduced saturated (SFAs) and increased unsaturated fatty acids (UFAs) from CPO supplementation. Supplementation of CPO improved some health indices, especially the AI, TI, and h:H indices. Fatty acid profiles from beef from heifers fed different CPO levels were successfully differentiated by chemometric analysis. Fatty acid composition alterations suggest consumer health benefits. These results show that CPO in the feed can make beef more nutritious by altering its fatty acid content. Further research, however, would be necessary to optimize CPO supplementation levels and to assess, if any, possible negative effects on meat quality or production efficiency for a long-term study.

## Figures and Tables

**Table 1:** Fatty acid profiles (% of total FAs) of cassava bioethanol by-product (LCEP and HCEP), crude palm oil (CPO), and rice straw (RS).

Fatty Acids	LCEP	HCEP	CPO	RS
C8:0	2.05	1.91	ND	ND
C10:0	1.83	1.94	ND	ND
C12:0	18.40	21.65	ND	ND
C14:0	8.21	9.39	1.05	1.28
C14:1	0.00	0.07	ND	ND
C16:0	22.00	24.96	45.31	47.49
C16:1	8.52	6.37	ND	ND
C18:0	3.20	3.63	3.95	8.57
C18:1n9t	0.00	0.30	37.72	ND
C18:1n9c	23.73	22.54	ND	16.76
C18:2n6c	11.17	6.55	10.75	19.88
C18:3n3	0.73	0.58	ND	6.03
C20:3n6	0.15	0.12	ND	ND
Total	100.00	100.00	100.00	100.00

FAs, fatty acids; LCEP, low level of cassava bioethanol by-product; HCEP, high level of cassava bioethanol by-product; CPO, crude palm oil; RS, rice straw; ND, not detected. The dietary ingredients and chemical composition of experimental diets are detailed in Table 1 and Table 2 of [17].

**Table 2:** Fatty acid profile (% of total FAs) of LL meat of beef heifer fed with different diets.

Fatty Acid	Cassava Bioethanol By-Product (CEP)		*p*-Value	Crude Palm Oil (CPO)			*p*-Value
	LCEP (15%)	HCEP (30%)		CPO-0 (0%)	CPO-2 (2%)	CPO-4 (4%)	
C12:0	0.37 ± 0.11	0.37 ± 0.11	NS	0.48 ± 0.01 ^a^	0.39 ± 0.00 ^b^	0.23 ± 0.01 ^c^	***
C13:0	0.25 ± 0.17	0.25 ± 0.17	NS	0.04 ± 0.03 ^c^	0.26 ± 0.01 ^b^	0.44 ± 0.02 ^a^	***
C14:0	4.99 ± 1.14	4.99 ± 1.12	NS	6.11 ± 0.12 ^a^	5.31 ± 0.04 ^b^	3.56 ± 0.07 ^c^	***
C14:1	0.45 ± 0.11	0.46 ± 0.10	NS	0.55 ± 0.06 ^a^	0.49 ± 0.03 ^a^	0.33 ± 0.00 ^b^	***
C16:0	28.07 ± 2.92	28.09 ± 2.86	NS	30.69 ± 0.27 ^a^	29.22 ± 0.11 ^b^	24.34 ± 0.42 ^c^	***
C16:1	14.41 ± 5.29	14.34 ± 4.88	NS	10.49 ± 0.92 ^b^	11.71 ± 0.20 ^b^	20.91 ± 2.05 ^a^	***
C18:0	16.47 ± 1.99	16.40 ± 1.88	NS	18.29 ± 0.71 ^a^	16.99 ± 0.38 ^b^	14.03 ± 0.09 ^c^	***
C18:1n9t	0.96 ± 0.54	0.95 ± 0.47	NS	1.39 ± 0.11 ^a^	1.10 ± 0.40 ^a^	0.37 ± 0.02 ^b^	***
C18:1n9c	29.03 ± 1.66	29.16 ± 1.08	NS	28.67 ± 0.10 ^b^	30.28 ± 0.80 ^a^	28.32 ± 1.74 ^b^	*
C18:2n6c	2.32 ± 0.82	2.32 ± 0.80	NS	1.60 ± 0.03 ^c^	1.98 ± 0.04 ^b^	3.38 ± 0.15 ^a^	***
C18:3n3	0.17 ± 0.09	0.18 ± 0.05	NS	0.15 ± 0.04	0.23 ± 0.00	0.16 ± 0.10	NS
C20:3n6	0.30 ± 0.16	0.31 ± 0.16	NS	0.18 ± 0.00 ^c^	0.22 ± 0.02 ^b^	0.52 ± 0.01 ^a^	***
C20:3n3	1.49 ± 0.65	1.50 ± 0.64	NS	0.92 ± 0.04 ^c^	1.21 ± 0.03 ^b^	2.34 ± 0.04 ^a^	***
C22:6n3	0.65 ± 0.28	0.64 ± 0.27	NS	0.38 ± 0.03 ^c^	0.55 ± 0.00 ^b^	1.01 ± 0.06 ^a^	***
SFAs	50.16 ± 5.95	50.11 ± 5.80	NS	55.62 ± 1.13 ^a^	52.18 ± 0.24 ^b^	42.61 ± 0.40 ^c^	***
MUFAs	44.87 ± 4.06	44.91 ± 3.91	NS	41.12 ± 0.98 ^c^	43.60 ± 0.15 ^b^	49.95 ± 0.35 ^a^	***
PUFAs	4.95 ± 1.90	4.96 ± 1.88	NS	3.25 ± 0.14 ^c^	4.21 ± 0.08 ^b^	7.42 ± 0.05 ^a^	***
Omega 3	2.32 ± 0.93	2.33 ± 0.92	NS	1.46 ± 0.11 ^c^	2.00 ± 0.01 ^b^	3.52 ± 0.08 ^a^	***
Omega 6	2.63 ± 0.98	2.63 ± 0.96	NS	1.78 ± 0.02 ^c^	2.20 ± 0.06 ^b^	3.90 ± 0.14 ^a^	***
Omega 6:3	1.15 ± 0.10	1.14 ± 0.06	NS	1.23 ± 0.08 ^a^	1.10 ± 0.02 ^b^	1.11 ± 0.06 ^b^	*
PUFA:SFA	0.10 ± 0.03	0.10 ± 0.03	NS	0.07 ± 0.00 ^c^	0.09 ± 0.00 ^b^	0.14 ± 0.00 ^a^	***
MUFA:SFA	0.91 ± 0.2	0.91 ± 0.19	NS	0.74 ± 0.03 ^c^	0.83 ± 0.00 ^b^	1.17 ± 0.01 ^a^	***
UFA:SFA	1.02 ± 0.25	1.02 ± 0.24	NS	0.79 ± 0.03 ^c^	0.91 ± 0.00 ^b^	1.34 ± 0.02 ^a^	***
HFA:SFA	0.66 ± 0.00	0.66 ± 0.00	NS	0.67 ± 0.00 ^a^	0.66 ± 0.00 ^a^	0.65 ± 0.00 ^b^	*
AI	0.99 ± 0.26	0.99 ± 0.25	NS	1.25 ± 0.04 ^a^	1.06 ± 0.00 ^b^	0.67 ± 0.01 ^c^	***
TI	1.63 ± 0.44	1.62 ± 0.42	NS	2.07 ± 0.11 ^a^	1.72 ± 0.01 ^b^	1.09 ± 0.00 ^c^	***
h:H	1.07 ± 0.17	1.07 ± 0.17	NS	0.90 ± 0.01 ^c^	1.03 ± 0.00 ^b^	1.29 ± 0.03 ^a^	***
NVI	1.66 ± 0.08	1.66 ± 0.08	NS	1.57 ± 0.00 ^c^	1.65 ± 0.00 ^b^	1.75 ± 0.03 ^a^	***
HPI	1.07 ± 0.31	1.07 ± 0.30	NS	0.79 ± 0.03 ^c^	0.94 ± 0.00 ^b^	1.47 ± 0.03 ^a^	***
PI	12.62 ± 4.81	12.63 ± 4.74	NS	8.24 ± 0.48 ^c^	10.82 ± 0.10 ^b^	18.8 ± 0.33 ^a^	***
SCD-16	33.38 ± 10.05	33.30 ± 9.50	NS	25.42 ± 1.84 ^b^	28.61 ± 0.44 ^b^	46.00 ± 2.87 ^a^	***
SCD-18	63.86 ± 2.85	64.04 ± 2.56	NS	61.08 ± 0.83 ^c^	64.04 ± 1.13 ^b^	66.74 ± 1.52 ^a^	***
EI	51.70 ± 3.02	51.77 ± 2.54	NS	53.27 ± 0.82 ^a^	53.59 ± 0.27 ^a^	48.34 ± 1.87 ^b^	***
TE	5.78 ± 0.81	5.78 ± 0.81	NS	5.02 ± 0.05 ^c^	5.50 ± 0.02 ^b^	6.83 ± 0.01 ^a^	***

^a,b,c^ Regarding the effects of CPO, values in the same row with different superscripts indicate significant differences (*p* < 0.05). FAs, fatty acids; LCEP, low level of cassava bioethanol by-product (15%); HCEP, high level of cassava bioethanol by-product (30%); CPO, crude palm oil (0, 2, and 4%); Int., interaction; * *p* < 0.05, *** *p* < 0.001, NS, non-significant.

**Table 3:** Fatty acid profile (% of total FAs) of SM meat of beef heifer fed with different diets.

Fatty Acid	Cassava Bioethanol By-Product (CEP)		*p*-Value	Crude Palm Oil (CPO)			*p*-Value
	LCEP (15%)	HCEP (30%)		CPO-0 (0%)	CPO-2 (2%)	CPO-4 (4%)	
C12:0	0.32 ± 0.08	0.32 ± 0.05	NS	0.33 ± 0.04 ^a^	0.25 ± 0.04 ^b^	0.38 ± 0.06 ^a^	**
C13:0	0.43 ± 0.07	0.43 ± 0.07	NS	0.46 ± 0.00 ^b^	0.50 ± 0.00 ^a^	0.33 ± 0.03 ^c^	***
C14:0	3.83 ± 0.60	3.82 ± 0.37	NS	4.11 ± 0.22 ^a^	3.37 ± 0.50 ^b^	3.99 ± 0.35 ^a^	*
C14:1	0.41 ± 0.03	0.41 ± 0.02	NS	0.44 ± 0.01 ^a^	0.38 ± 0.01 ^b^	0.43 ± 0.03 ^a^	**
C16:0	24.55 ± 1.53	24.6 ± 0.95	NS	25.19 ± 0.05 ^a^	23.43 ± 1.61 ^b^	25.1 ± 0.49 ^a^	*
C16:1	20.44 ± 3.11	20.25 ± 2.11	NS	18.23 ± 0.78 ^b^	22.66 ± 3.18 ^a^	20.13 ± 0.34 ^a,b^	*
C18:0	13.55 ± 1.22	13.62 ± 0.77	NS	13.53 ± 0.00 ^a,b^	12.82 ± 1.36 ^b^	14.4 ± 0.01 ^a^	*
C18:1n9t	0.47 ± 0.08	0.47 ± 0.06	NS	0.44 ± 0.06 ^b^	0.42 ± 0.01 ^b^	0.55 ± 0.03 ^a^	***
C18:1n9c	27.86 ± 1.26	27.94 ± 0.98	NS	28.73 ± 1.22 ^a^	28.20 ± 0.04 ^a^	26.77 ± 0.38 ^b^	**
C18:2n6c	3.60 ± 0.31	3.57 ± 0.20	NS	3.73 ± 0.32 ^a^	3.36 ± 0.16 ^b^	3.66 ± 0.01 ^a^	*
C18:3n3	0.23 ± 0.10	0.23 ± 0.08	NS	0.27 ± 0.01 ^a^	0.12 ± 0.08 ^b^	0.30 ± 0.00 ^a^	***
C20:3n6	0.54 ± 0.03	0.54 ± 0.01	NS	0.55 ± 0.00	0.53 ± 0.00	0.54 ± 0.04	NS
C20:3n3	2.58 ± 0.35	2.59 ± 0.23	NS	2.70 ± 0.25 ^a^	2.73 ± 0.21 ^a^	2.31 ± 0.21 ^b^	*
C22:6n3	1.14 ± 0.12	1.13 ± 0.09	NS	1.21 ± 0.01 ^a^	1.17 ± 0.11 ^a^	1.03 ± 0.03 ^b^	**
SFAs	42.69 ± 3.29	42.81 ± 2.00	NS	43.65 ± 0.32 ^a^	40.39 ± 3.54 ^b^	44.23 ± 0.93 ^a^	*
MUFAs	49.19 ± 3.07	49.08 ± 2.02	NS	47.85 ± 0.40 ^b^	51.66 ± 3.10 ^a^	47.90 ± 0.65 ^b^	*
PUFAs	8.10 ± 0.47	8.09 ± 0.33	NS	8.49 ± 0.07 ^a^	7.93 ± 0.43 ^b^	7.86 ± 0.28 ^b^	*
Omega 3	3.95 ± 0.40	3.97 ± 0.27	NS	4.19 ± 0.24 ^a^	4.04 ± 0.25 ^a^	3.65 ± 0.25 ^b^	*
Omega 6	4.14 ± 0.32	4.12 ± 0.21	NS	4.29 ± 0.32 ^a^	3.89 ± 0.17 ^b^	4.21 ± 0.02 ^a^	*
Omega 6:3	1.06 ± 0.14	1.05 ± 0.09	NS	1.03 ± 0.14 ^a,b^	0.96 ± 0.01 ^b^	1.16 ± 0.07 ^a^	*
PUFA:SFA	0.16 ± 0.01	0.16 ± 0.01	NS	0.17 ± 0.00 ^a^	0.15 ± 0.00 ^c^	0.16 ± 0.00 ^b^	***
MUFA:SFA	1.16 ± 0.18	1.15 ± 0.10	NS	1.09 ± 0.01 ^b^	1.30 ± 0.19 ^a^	1.08 ± 0.03 ^b^	*
UFA:SFA	1.36 ± 0.21	1.34 ± 0.11	NS	1.29 ± 0.01 ^b^	1.50 ± 0.22 ^a^	1.26 ± 0.04 ^b^	*
HFA:SFA	0.67 ± 0.00	0.67 ± 0.00	NS	0.67 ± 0.00 ^a^	0.67 ± 0.00 ^a^	0.66 ± 0.00 ^b^	**
AI	0.70 ± 0.10	0.70 ± 0.06	NS	0.74 ± 0.02 ^a^	0.63 ± 0.09 ^b^	0.74 ± 0.04 ^a^	*
TI	1.07 ± 0.13	1.06 ± 0.08	NS	1.08 ± 0.03 ^a,b^	0.97 ± 0.14 ^b^	1.15 ± 0.05 ^a^	*
h:H	1.29 ± 0.13	1.28 ± 0.07	NS	1.28 ± 0.04 ^a,b^	1.37 ± 0.12 ^b^	1.21 ± 0.05 ^a^	*
NVI	1.71 ± 0.08	1.71 ± 0.05	NS	1.69 ± 0.04 ^b^	1.77 ± 0.06 ^a^	1.66 ± 0.04 ^b^	*
HPI	1.44 ± 0.25	1.42 ± 0.14	NS	1.34 ± 0.03 ^b^	1.62 ± 0.26 ^a^	1.34 ± 0.08 ^b^	*
PI	20.69 ± 1.60	20.66 ± 1.11	NS	21.72 ± 0.27	20.85 ± 1.48	19.45 ± 0.79	*
SCD-16	45.22 ± 5.00	44.94 ± 3.31	NS	41.94 ± 0.99 ^b^	48.79 ± 5.21 ^a^	44.52 ± 0.91 ^a,b^	*
SCD-18	67.30 ± 2.55	67.25 ± 1.87	NS	67.91 ± 0.93 ^a^	68.90 ± 2.28 ^a^	65.00 ± 0.30 ^b^	**
EI	47.93 ± 1.82	48.10 ± 1.20	NS	49.31 ± 1.20 ^a^	47.09 ± 1.70 ^b^	47.64 ± 0.32 ^a,b^	*
TE	6.51 ± 0.74	6.46 ± 0.42	NS	6.13 ± 0.31 ^b^	7.01 ± 0.61 ^a^	6.31 ± 0.43 ^a,b^	*

^a,b,c^ Regarding the effects of CPO, values in the same row with different superscripts indicate significant differences (*p* < 0.05). FAs, fatty acids; LCEP, low level of cassava bioethanol by-product (15%); HCEP, high level of cassava bioethanol by-product (30%); CPO, crude palm oil (0, 2, and 4%); Int., interaction; * *p* < 0.05, ** *p* < 0.01, *** *p* <0.001, NS, non-significant.

**Table 4:** Fatty acid profile (% of FAs) of subcutaneous fat of beef heifer fed with different diets.

Treatment	LCEP			HCEP			*p*-Value		
	CPO-0	CPO-2	CPO-4	CPO-0	CPO-2	CPO-4	CEP	CPO	Int.
C10:0	0.10 ± 0.02 ^A,a^	0.06 ± 0.01 ^A,b^	0.06 ± 0.01 ^A,b^	0.07 ± 0.01 ^B,a^	0.05 ± 0.03 ^B,b^	0.05 ± 0.01 ^B,b^	*	***	NS
C12:0	1.00 ± 0.09	0.98 ± 0.29	1.05 ± 0.03	1.05 ± 0.04	1.07 ± 0.27	1.27 ± 0.35	NS	NS	NS
C13:0	0.16 ± 0.03 ^a^	0.11 ± 0.04 ^b^	0.14 ± 0.03 ^b^	0.20 ± 0.03 ^a^	0.14 ± 0.02 ^b^	0.13 ± 0.01 ^b^	NS	**	NS
C14:0	10.54 ± 0.59	9.89 ± 0.65	9.95 ± 0.34	10.4 ± 0.21	10.18 ± 0.64	9.39 ± 0.53	NS	NS	NS
C16:0	36.35 ± 0.16 ^a,b^	35.85 ± 1.57 ^b^	37.37 ± 0.42 ^a^	36.29 ± 0.13 ^a,b^	35.64 ± 0.55 ^b^	36.78 ± 0.47 ^a^	NS	*	NS
C16:1	0.80 ± 0.77 ^y^	1.89 ± 0.04 ^x^	1.70 ± 0.15 ^x^	2.13 ± 0.03 ^x^	2.07 ± 0.08 ^x^	2.23 ± 0.18 ^x^	***	*	*
C18:0	22.02 ± 3.58	20.29 ± 0.90	21.84 ± 0.94	19.98 ± 6.33	19.44 ± 1.62	22.45 ± 5.19	NS	NS	NS
C18:1n9c	28.30 ± 1.68	30.15 ± 3.52	27.19 ± 0.04	28.84 ± 5.99	30.38 ± 3.08	26.82 ± 6.19	NS	NS	NS
C18:2n6t	0.16 ± 0.03 ^a^	0.10 ± 0.03 ^b^	0.07 ± 0.01 ^b^	0.16 ± 0.05 ^a^	0.10 ± 0.02 ^b^	0.10 ± 0.07 ^b^	NS	*	NS
C18:2n6c	0.24 ± 0.20 ^y^	0.43 ± 0.05 ^x^	0.41 ± 0.01 ^x^	0.52 ± 0.03 ^x^	0.50 ± 0.01 ^x^	0.42 ± 0.01 ^x^	**	NS	*
C9T11	0.11 ± 0.02 ^B^	0.09 ± 0.02 ^B^	0.11 ± 0.01 ^B^	0.14 ± 0.02 ^A^	0.13 ± 0.02 ^A^	0.11 ± 0.02 ^A^	**	NS	NS
C18:3n3	0.10 ± 0.07 ^B^	0.08 ± 0.07 ^B^	0.02 ± 0.01 ^B^	0.08 ± 0.06 ^A^	0.13 ± 0.03 ^A^	0.12 ± 0.04 ^A^	*	NS	NS
C9C11	0.01 ± 0.00 ^B^	0.01 ± 0.00 ^B^	0.01 ± 0.00 ^B^	0.01 ± 0.01 ^A^	0.03 ± 0.02 ^A^	0.01 ± 0.01 ^A^	*	NS	NS
T10C12	0.03 ± 0.01	0.02 ± 0.01	0.03 ± 0.01	0.03 ± 0.01	0.03 ± 0.01	0.03 ± 0.01	NS	NS	NS
C21:0	0.04 ± 0.01 ^B^	0.05 ± 0.02 ^B^	0.05 ± 0.01 ^B^	0.06 ± 0.02 ^A^	0.08 ± 0.01 ^A^	0.07 ± 0.02 ^A^	*	NS	NS
C20:2	0.03 ± 0.01	0.03 ± 0.01	0.03 ± 0.01	0.03 ± 0.01	0.03 ± 0.01	0.03 ± 0.01	NS	NS	NS
C20:3n6	0.02 ± 0.01 ^y,z^	0.04 ± 0.02 ^x^	0.01 ± 0.01 ^z^	0.02 ± 0.01 ^y,z^	0.03 ± 0.01 ^y^	0.02 ± 0.01 ^y,z^	NS	**	*
C20:3n3	0.04 ± 0.02 ^a^	0.03 ± 0.01 ^b^	0.02 ± 0.01 ^b^	0.04 ± 0.01 ^a^	0.03 ± 0.01 ^b^	0.03 ± 0.01 ^b^	NS	*	NS
C22:6n3	0.02 ± 0.01 ^B,a^	0.00 ± 0.00 ^B,b^	0.03 ± 0.01 ^B,a^	0.03 ± 0.01 ^A,a^	0.02 ± 0.02 ^A,b^	0.04 ± 0.01 ^A,a^	*	**	NS
SFAs	70.19 ± 2.77	67.2 ± 3.45	70.43 ± 0.14	68.03 ± 5.99	66.59 ± 3.06	70.12 ± 6.50	NS	NS	NS
MUFAs	29.10 ± 2.45	32.03 ± 3.56	28.89 ± 0.11	30.97 ± 6.02	32.44 ± 3.00	29.04 ± 6.37	NS	NS	NS
PUFAs	0.73 ± 0.32 ^B^	0.78 ± 0.12 ^B^	0.69 ± 0.03 ^B^	1.01 ± 0.04 ^A^	0.98 ± 0.06 ^A^	0.86 ± 0.14 ^A^	***	NS	NS
Omega 3	0.15 ± 0.09 ^B^	0.10 ± 0.07 ^B^	0.06 ± 0.01 ^B^	0.14 ± 0.06 ^A^	0.18 ± 0.05 ^A^	0.18 ± 0.03 ^A^	*	NS	NS
Omega 6	0.59 ± 0.24 ^B^	0.69 ± 0.06 ^B^	0.64 ± 0.02 ^B^	0.88 ± 0.09 ^A^	0.81 ± 0.02 ^A^	0.68 ± 0.11 ^A^	*	NS	NS
Omega 6:3	4.64 ± 1.01 ^y^	11.29 ± 6.60 ^x^	11.83 ± 0.44 ^x^	8.22 ± 4.22 ^x,y^	5.02 ± 1.21 ^y^	3.90 ± 0.04 ^y^	*	NS	*
PUFA:SFA	0.01 ± 0.01 ^B^	0.01 ± 0.01 ^B^	0.01 ± 0.01 ^B^	0.02 ± 0.01 ^A^	0.02 ± 0.01 ^A^	0.02 ± 0.01 ^A^	*	NS	NS
MUFA:SFA	0.42 ± 0.06	0.48 ± 0.08	0.41 ± 0.01	0.47 ± 0.13	0.49 ± 0.07	0.43 ± 0.14	NS	NS	NS
UFA:SFA	0.43 ± 0.06	0.49 ± 0.08	0.42 ± 0.01	0.48 ± 0.14	0.51 ± 0.07	0.44 ± 0.14	NS	NS	NS
HFA:SFA	0.68 ± 0.04	0.70 ± 0.01	0.69 ± 0.02	0.71 ± 0.07	0.70 ± 0.02	0.68 ± 0.05	NS	NS	NS
AI	2.68 ± 0.17	2.37 ± 0.39	2.64 ± 0.05	2.55 ± 0.45	2.34 ± 0.32	2.68 ± 0.68	NS	NS	NS
TI	4.52 ± 0.67	3.98 ± 0.54	4.61 ± 0.04	4.28 ± 1.21	3.81 ± 0.54	4.68 ± 1.39	NS	NS	NS
h:H	0.62 ± 0.04	0.58 ± 0.11	0.59 ± 0.01	0.64 ± 0.13	0.69 ± 0.09	0.60 ± 0.15	NS	NS	NS
NVI	1.38 ± 0.06	1.41 ± 0.14	1.31 ± 0.05	1.35 ± 0.02	1.40 ± 0.07	1.34 ± 0.05	NS	NS	NS
HPI	0.37 ± 0.03	0.43 ± 0.08	0.38 ± 0.01	0.40 ± 0.08	0.43 ± 0.06	0.40 ± 0.11	NS	NS	NS
PI	1.71 ± 0.49 ^B^	1.71 ± 0.10 ^B^	1.61 ± 0.06 ^B^	2.09 ± 0.18 ^A^	2.09 ± 0.28 ^A^	1.96 ± 0.30 ^A^	*	NS	NS
SCD-16	2.10 ± 2.02 ^y^	5.00 ± 0.31 ^x^	4.34 ± 0.31 ^x^	5.54 ± 0.06 ^x^	5.48 ± 0.12 ^x^	5.71 ± 0.50 ^x^	***	*	*
SCD-18	56.45 ± 5.47	59.58 ± 3.89	55.49 ± 1.04	59.17 ± 12.68	60.86 ± 4.40	54.21 ± 11.46	NS	NS	NS
EI	57.51 ± 1.53	57.18 ± 2.27	55.65 ± 0.85	55.97 ± 0.27	56.91 ± 1.13	55.81 ± 0.68	NS	NS	NS
TE	0.008 ± 0.00 ^B^	0.014 ± 0.00 ^B^	0.015 ± 0.01 ^B^	0.018 ± 0.00 ^A^	0.016 ± 0.00 ^A^	0.16 ± 0.00 ^A^	*	NS	NS

^A,B^ Values in the same row with different superscripts indicate significant differences in the effect of CEP. ^a,b^ Values in the same row with different superscripts indicate significant differences in the effect of CPO. ^x,y,z^ Values in the same row with different superscripts indicate significant differences in the effect of CEP × CPO combination. FAs, fatty acids; LCEP, low level of cassava bioethanol by-product (15%); HCEP, high level of cassava bioethanol by-product (30%); CPO, crude palm oil (0, 2, and 4%); Int., interaction; * *p* < 0.05, ** *p* < 0.01, *** *p* <0.001, NS, non-significant.

## Data Availability

Data are contained within the article.

## Supplementary Materials

The following supporting information can be downloaded at: https://www.mdpi.com/article/10.3390/ani14233478/s1, Figures S1–S3: PCA, OPLS-DA, and permutation test of CPO-0 vs. CPO-2, CPO-0 vs. CPO-4, and CPO-2 vs. CPO-4 in LL, SM, and fat; Table S1: Possible biomarker for separating fatty acid profiles from different levels of crude palm oil.
