# Supplementary material for: Effect of Cassava Bioethanol By-Products and Crude Palm Oil Feeding on Fatty Acid Composition of Beef Meat and Fat in Crossbred Thai Indigenous Heifers
Source: Animals (Basel). 2024 Dec 2;14(23):3478. doi: 10.3390/ani14233478 (PMC11640199; doi:10.3390/ani14233478)
Supplement: Supplementary file 1 [file animals-14-03478-s001.zip › animals-3331054-supplementary.pdf]

Table S1. Possible biomarker for separating fatty acid profiles from different levels of crude palm oil.

Item	VIP	*p*-Value	-log10(p)	FDR	R^2X	R^2Y	Q^2
CPO-0 vs CPO-2							
Longissimus et lumborum (LL)							
C18:2n6c	1.145	1.4E-08	7.866	1.33E-07	0.82	1.00	0.98
C13:0	1.144	1.9E-08	7.721	1.33E-07			
C14:0	1.139	4.3E-08	7.362	2.03E-07			
C20:3n3	1.138	6.2E-08	7.209	2.09E-07			
C12:0	1.137	7.5E-08	7.127	2.09E-07			
C22:6n3	1.129	2.3E-07	6.630	5.42E-07			
C16:0	1.128	2.7E-07	6.567	5.42E-07			
Semimembranosus (SM)							
C13:0	1.506	1.86E-06	5.730	2.61E-05	0.79	1.00	0.98
C14:1	1.424	1.20E-04	3.921	8.40E-04			
C20:3n6	1.309	5.44E-04	3.265	2.54E-03			
C18:3n3	1.264	1.33E-03	2.878	4.64E-03			
C16:1	1.159	7.89E-03	2.103	2.05E-02			
C14:0	1.101	8.78E-03	2.057	2.05E-02			
C12:0	1.034	1.32E-02	1.880	2.64E-02			
C16:0	1.023	2.31E-02	1.636	0.040			
Subcutaneous fat (Fat)							
C18:2n6t	1.697	2.28E-03	2.642	4.33E-02	0.63	1.00	0.94
CPO-0 vs CPO-4							
Longissimus et lumborum (LL)							
C20:3n3	1.093	5.2E-14	13.284	4.07E-13	0.97	1.00	1.00
C20:3n6	1.093	5.8E-14	13.236	4.07E-13			
C14:0	1.091	1.1E-12	11.957	5.15E-12			
C16:0	1.088	3.1E-11	10.510	9.80E-11			
C12:0	1.088	3.5E-11	10.456	9.80E-11			
C18:2n6c	1.087	1.1E-10	9.960	2.56E-10			
C13:0	1.086	1.4E-10	9.849	2.83E-10			
C22:6n3	1.083	8.6E-10	9.067	1.50E-09			
C18:1n9t	1.082	1.1E-09	8.947	1.76E-09			
C18:0	1.069	5.0E-08	7.299	7.03E-08			
C16:1	1.054	5.1E-07	6.296	6.44E-07			
C14:1	1.028	5.6E-06	5.250	6.57E-06			
Semimembranosus (SM)							
C18:0	1.517	4.53E-18	17.344	6.34E-17	1.00	1.00	1.00
C22:6n3	1.469	2.49E-07	6.604	1.74E-06			
C13:0	1.446	1.68E-06	5.776	7.82E-06			
C16:1	1.313	2.80E-04	3.553	9.80E-04			
C18:1n9t	1.171	3.29E-03	2.483	8.86E-03			
C18:1n9c	1.160	3.80E-03	2.421	8.86E-03			

Item	VIP	*p*-Value	-log10(p)	FDR	R^2X	R^2Y	Q^2
C18:3n3	1.089	8.59E-03	2.066	1.72E-02			
C20:3n3	1.022	1.64E-02	1.786	2.87E-02			
Subcutaneous fat (Fat)							
-	-	-	-	-	-	-	-
CPO-2 vs CPO-4							
Longissimus et lumborum (LL)							
C13:0	1.506	1.86E-06	5.730	0.000	0.79	1.00	0.98
C14:1	1.424	1.20E-04	3.921	0.001			
C20:3n6	1.309	5.44E-04	3.265	0.003			
C18:3n3	1.264	1.33E-03	2.878	0.005			
C16:1	1.159	7.89E-03	2.103	0.020			
C14:0	1.101	8.78E-03	2.057	0.020			
C12:0	1.034	1.32E-02	1.880	0.026			
C16:0	1.023	2.31E-02	1.636	0.040			
Semimembranosus (SM)							
C13:0	1.304	1.07E-07	6.972	1.49E-06	0.89	1.00	0.99
C18:1n9c	1.278	3.82E-06	5.418	2.67E-05			
C18:1n9t	1.247	1.88E-05	4.726	8.77E-05			
C18:3n3	1.145	3.81E-04	3.419	1.33E-03			
C18:2n6c	1.097	1.44E-03	2.841	4.04E-03			
C12:0	1.045	3.24E-03	2.489	7.56E-03			
C14:1	1.030	4.22E-03	2.375	8.44E-03			
Subcutaneous fat (Fat)							
-	-	-	-	-	-	-	-

VIP, variable importance in projection; FDR, false recovery rate

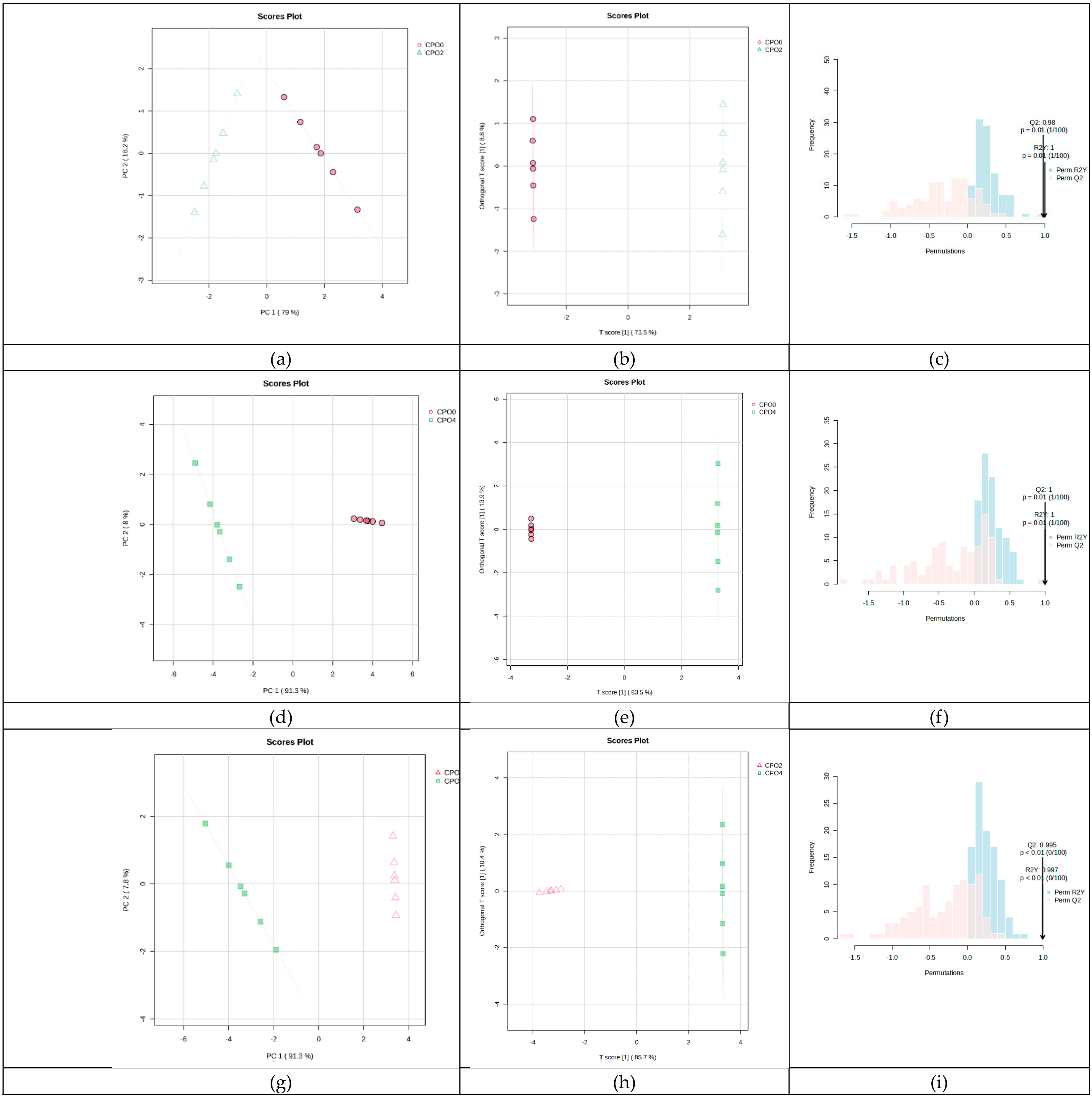

Figure S1. PCA (a, d, g), OPLS-DA (b, e, h), and permutation test (c, f, i) of CPO-0 vs CPO-2, CPO-0 vs CPO-4, and CPO-2- vs CPO-4 in LL meat.

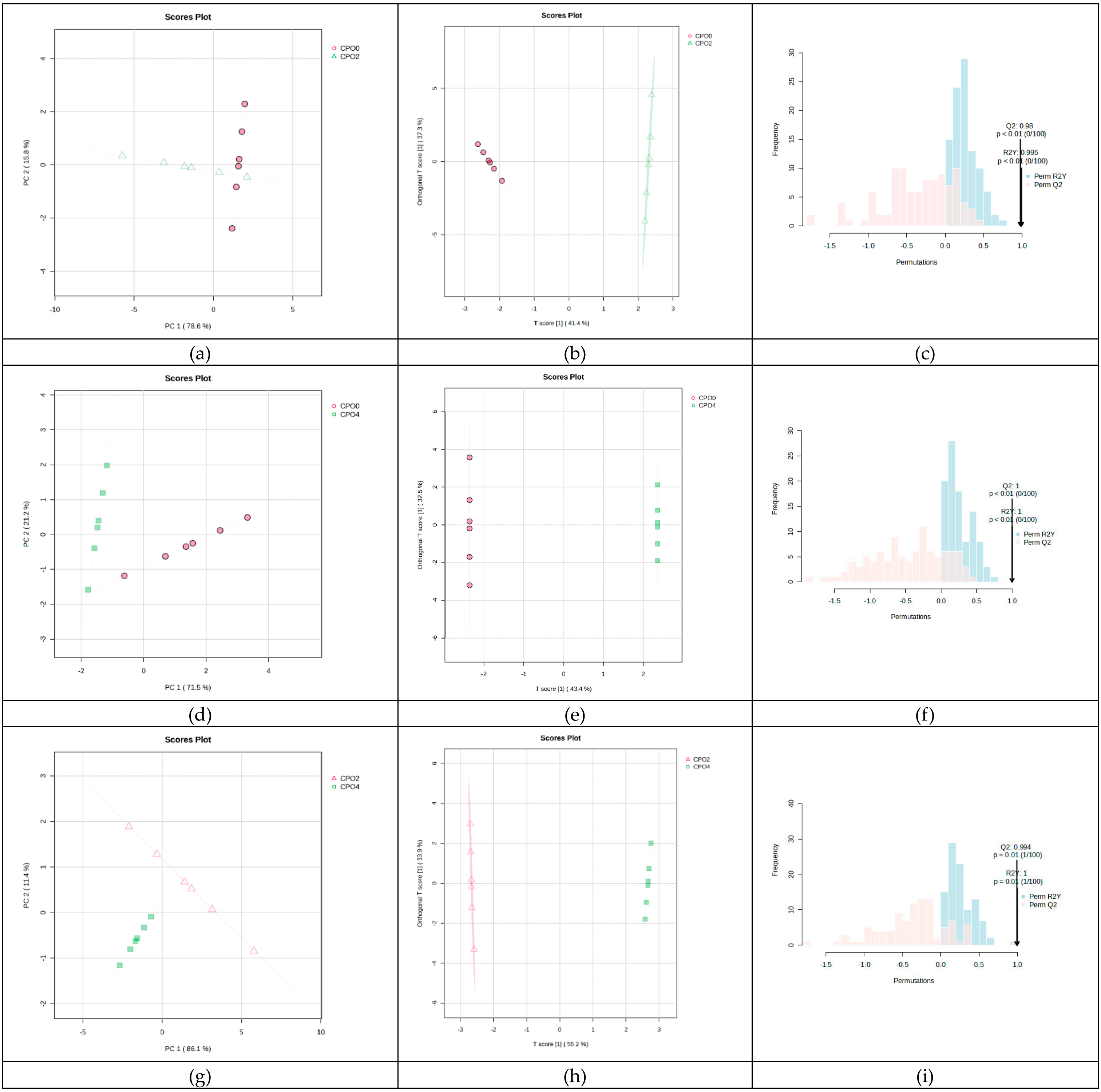

Figure S2. PCA (a, d, g), OPLS-DA (b, e, h), and permutation test (c, f, i) of CPO-0 vs CPO-2, CPO-0 vs CPO-4, and CPO-2- vs CPO-4 in SM meat.

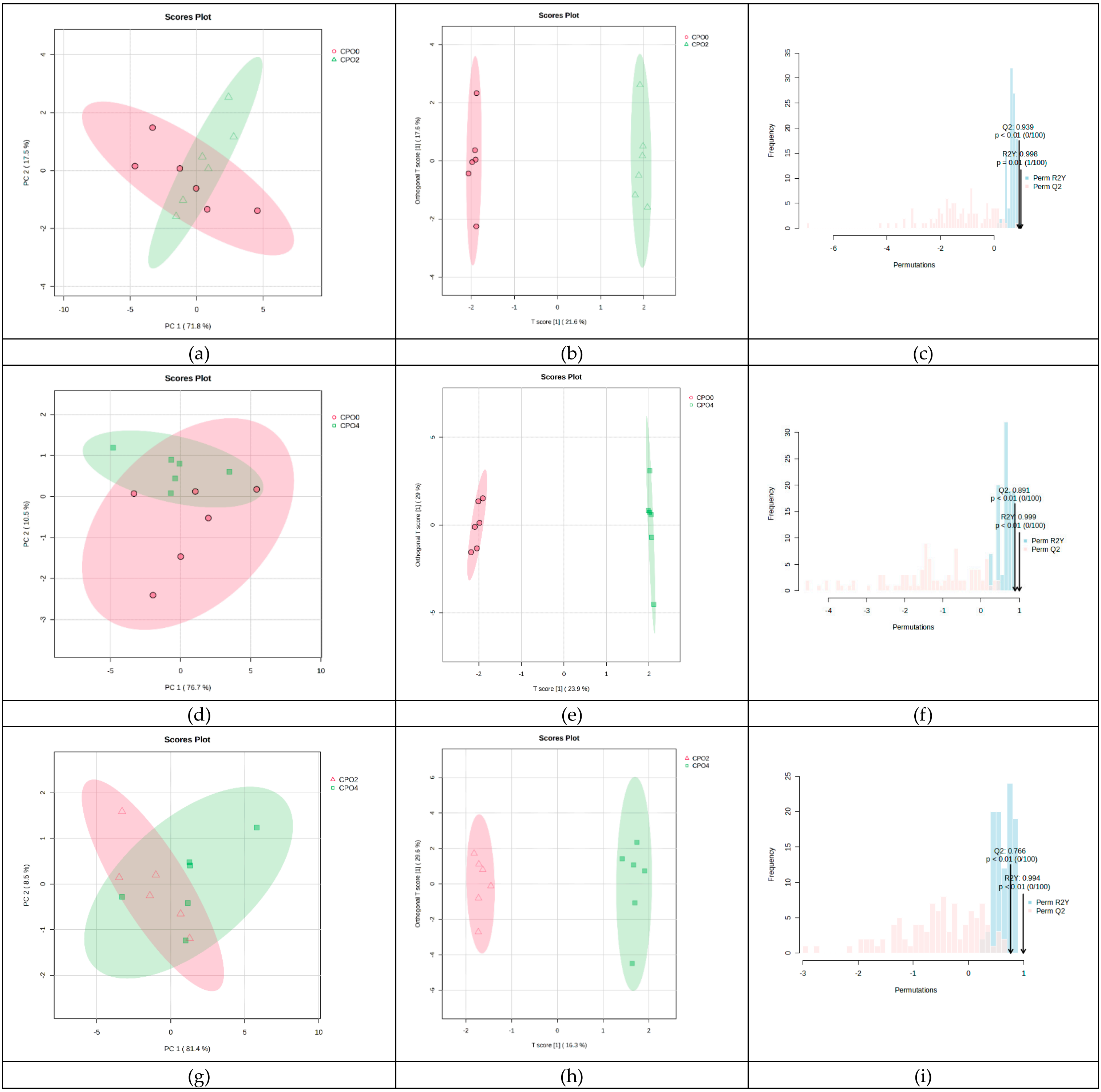

Figure S3. PCA (a, d, g), OPLS-DA (b, e, h), and permutation test (c, f, i) of CPO-0 vs CPO-2, CPO-0 vs CPO-4, and CPO-2- vs CPO-4 in subcutaneous fat.
